# Navigating the orbital complications of endoscopic sinus surgery: a systematic review of 204,286 patients

**DOI:** 10.3389/fsurg.2026.1730239

**Published:** 2026-02-24

**Authors:** Feras Alkholaiwi, Laila Zamil Alzamil, Reuof Mohammed Alotaibi, Layan Ahmed Alrehaili, Nouf Saleh AlBlaihed, Joud Nasser Bindekhayel, Bushra Saud Bin Dalah, Shawq Fayez Aljabri, Reyouf Abdullah Aba Alhaweel, Anas Bassam Barnawi

**Affiliations:** 1 Department of Otorhinolaryngology, Head and Neck Surgery, College of Medicine, Imam Mohammad Ibn Saud Islamic University (IMSIU), Riyadh, Saudi Arabia; 2 College of Medicine, Imam Mohammad Ibn Saud Islamic University (IMSIU), Riyadh, Saudi Arabia

**Keywords:** orbital abscess, endoscopic sinus surgery, orbital cellulitis, orbital complications, orbital emphysema, orbital hematoma

## Abstract

**Background:**

The association between endoscopic sinus surgery and orbital complications is a complex and multifaceted one. While the precise relationship between the two is not fully elucidated, there is increasing evidence indicating a significant correlation between endoscopic sinus surgery and orbital complications.

**Objectives:**

To comprehensively examine the orbital complications that can arise from endoscopic sinus surgery, including their incidence, etiology, clinical manifestations, management strategies, and outcomes.

**Methods:**

An extensive search was conducted across multiple relevant databases to identify studies meeting the established inclusion criteria. The databases examined included PubMed, MEDLINE, and Embase, and the following search terms were used: (“Endoscopic sinus surgery” OR “FESS” OR “ESS”) AND (“Orbital complications” OR “Ocular injury” OR “Periorbital hemorrhage”). The search was limited to English-language publications from 2011 to 2023 and no restrictions were applied regarding study design during the search. Duplicate entries were removed, and the Rayyan QRCI tool was employed to streamline the selection and screening of studies. Due to heterogeneity in study designs, populations, and definitions of orbital complications among the included studies, a formal meta-analysis was not performed. Instead, a narrative synthesis was undertaken. The overall incidence of orbital complications was calculated by pooling the total number of reported complications across all included studies and dividing this by the total number of patients.

**Results:**

A total of eight studies, encompassing a combined population of 204,286 patients, were included in our final analysis. Of this population, 118,567 individuals (58%) were male. All patients underwent surgery for chronic rhinosinusitis. The reported orbital complication rates varied widely across studies, ranging from 0% in image-guided ESS to 27.6% in conventional ESS. Across the included studies, a total of 358 orbital complications were reported among 204,286 patients The most frequently reported orbital complications following sinus surgery were orbital injury, orbital hematoma, and orbital subcutaneous emphysema.

**Conclusion:**

ESS orbital complications are uncommon but have the potential to be dangerous. The available evidence suggests an association between the use of image-guided ESS and lower reported complication rates. Future prospective and randomized trials are required to determine the safest approach to ESS to avoid complications.

**Systematic Review Registration:**

Prospero CRD42024546806.

## Background

Endoscopic sinus surgery (ESS) is a common procedure used to treat chronic rhinosinusitis (CRS) and other nasal conditions. While ESS is generally safe and effective, potential complications can arise, particularly in the orbital region Orbital complications of ESS can be serious and require prompt recognition and management to prevent permanent damage to the eye and surrounding structures (1).

These complications can be classified into two main categories: intraoperative and postoperative. Intraoperative complications may include injury to the orbital contents, optic nerve, or extraocular muscles, as well as hemorrhage or infection in the orbital region. Postoperative complications, which develop in the days or weeks following the procedure, can include orbital hematoma, orbital cellulitis, orbital emphysema, and orbital abscess (2).

Orbital hematoma is one of the most dangerous orbital consequences of ESS. Orbital hematoma can cause proptosis, decreased vision, and pain. If left untreated, it can lead to permanent vision loss. Orbital cellulitis is another potentially serious complication of ESS, characterized by inflammation and infection of the orbital tissues (3, 4). Symptoms of orbital cellulitis may include redness and swelling of the eyelids, pain with eye movement, and fever. Prompt treatment with antibiotics and sometimes surgical drainage is necessary to prevent vision loss and other complications (3).

Orbital emphysema is a less common complication of ESS, but can still cause significant morbidity. This condition occurs when air is trapped within the orbit, usually as a result of communication between the nasal sinuses and the orbit (5). Symptoms of orbital emphysema may include crepitus (crackling sensation) around the eye, proptosis, and decreased vision. Treatment may involve observation, nasal decongestants, or surgical decompression (6).

The uncommon but potentially fatal orbital abscess is an ESS consequence and typically results from untreated orbital cellulitis or a direct infection spreading from the sinuses (7). Symptoms of orbital abscess may include severe eye pain, fever, proptosis, and limited eye movement. Immediate surgical drainage and intravenous antibiotics are necessary to prevent serious complications such as cavernous sinus thrombosis or meningitis (8).

Prompt identification and management of orbital complications are essential to preserving vision and preventing irreversible damage. When performing ESS, surgeons should be mindful of the possibility of orbital complications and take precautions, including cautious dissection and thorough hemostasis, to reduce risk. Patients with ESS should be informed about the warning signs and symptoms of orbital problems and advised to consult a physician if they materialize. Most orbital complications of ESS can be successfully treated with good patient outcomes if they are managed properly.

Understanding orbital complications of ESS is crucial for improving patient safety, optimizing surgical outcomes, and guiding clinical decision-making in this field. By conducting a systematic review, this study aims to consolidate existing knowledge and identify gaps in the literature for future projects.

## Study aim

The aim of undertaking this systematic review was to comprehensively examine orbital complications that can arise from endoscopic sinus surgery, including their incidence, etiology, clinical manifestations, management strategies, and outcomes.

### Study objectives

To estimate the incidence of orbital complications following ESS.

To identify the common risk factors that may provoke orbital complications in patients undergoing ESS.

To evaluate the clinical presentation and outcomes of orbital complications post-ESS.

To assess the effectiveness of different management approaches for orbital complications in this context.

## Methodology

This systematic review was conducted in strict adherence to the guidelines outlined by the Preferred Reporting Items for Systematic Reviews and Meta-Analyses (PRISMA) framework (9), ensuring a highly rigorous, transparent, and replicable approach to both data collection and reporting. To ensure a broad and comprehensive understanding of the research area, an extensive search was undertaken across several major electronic databases, including PubMed, Web of Science, SCOPUS, and ScienceDirect, targeting studies relevant to the topic under investigation. By limiting the selection to studies published in English, we aimed to maintain consistency in the quality of the research while also ensuring the accessibility and clarity of the included sources. This systematic approach was designed to provide a robust foundation for synthesizing evidence and drawing meaningful conclusions from available literature. Endoscopic Sinus Surgery (ESS) and Functional Endoscopic Sinus Surgery (FESS) are considered synonymous. The term “orbital complications” encompasses all adverse events involving the orbit or periorbital tissues related to ESS.

The search strategy was meticulously designed, utilizing a combination of carefully selected keywords and medical subject headings (MeSH) terms that specifically targeted the relationship between endoscopic sinus surgery and the occurrence of orbital complications. This ensured the retrieval of the most pertinent literature available.

Two independent reviewers carried out the screening process, which involved reviewing titles, abstracts, and full texts to determine the eligibility of each study. This dual-review process minimized potential biases and ensured the objectivity of the selection. Upon identifying the eligible studies, the reviewers extracted key data and performed a detailed evaluation of each study's methodological quality. To assess the robustness and reliability of the included research, established evaluation tools were applied, ensuring that only high-quality studies were incorporated into the final analysis. Discrepancies between reviewers were resolved through discussion or, when necessary, by consulting a third reviewer to ensure consensus.

## Inclusion criteria

Studies reporting on orbital complications following endoscopic sinus surgery.

Studies written or translated into the English language.

Studies with a clear description of orbital complications, including incidence, risk factors, clinical presentation, management, and outcomes.

Randomized controlled trials (RCTs), observational studies, cross-sectional studies, case-control studies, cohort studies, and case series.

## Exclusion criteria

Studies that looked at endoscopic nasal surgery for conditions affecting the eye area, skull base, or nasal tumors involving the eye.

Studies that were not about eye-related complications from endoscopic sinus surgery were excluded.

Animal experiments, lab-based studies, and review articles without new data were omitted.

Any studies with unclear or incomplete information about eye complications were not included.

Studies that focused on other types of complications, not related to the eye, after endoscopic sinus surgery, were excluded.

Case reports or expert opinions without strong evidence were also left out.

## Data extraction

To ensure the search results were accurate, we used Rayyan QCRI (10), a web tool that helps simplify systematic reviews. The titles and summaries of the studies found in the search were carefully checked to see if they matched our inclusion and exclusion criteria. Studies that fit the criteria were then reviewed in more detail by the research team. If there were any disagreements among reviewers, they discussed the issues to come to an agreement.

A structured data extraction process was implemented using a pre-designed template to capture essential information from each study. This included study titles, author names, publication year, geographical location, participant demographics, gender distribution, prevalence rates of ocular complications, reported risk factors, management strategies and the primary outcomes of interest. The template helped ensure consistency in data collection across studies. In addition, a separate document was created to systematically assess the potential for bias in the included studies.

## Data synthesis strategy

Summary tables were created to provide a clear qualitative assessment of the research findings, organizing key data such as study design, sample size, outcomes, and participant details. Given the substantial heterogeneity observed across the included studies regarding surgical techniques, study designs, sample sizes, and definitions of orbital complications, a formal meta-analysis to calculate a single pooled incidence rate was deemed inappropriate. Therefore, a narrative synthesis was conducted, as planned. This synthesis descriptively reports the range of complication rates, the absolute frequency of events, and qualitatively analyzes the potential sources of the observed variability, such as the impact of surgical technology (e.g., image guidance) and study methodology.

## Risk of bias assessment

The methodological quality of the included studies was assessed using the Methodological Index for Non-Randomized Studies (MINORS) tool. Overall, the included studies demonstrated moderate methodological quality. The detailed quality assessment results are presented in Table 1.

**Table 1:** Risk of bias assessment (MINORS).

Authors	Suzuki et al., 2015 (6)	Stankiewicz et al., 2011 (11)	Asaka et al., 2012 (12)	Seredyka-Burduk et al., 2017 (1)	Alharbi et al., 2023 (13)	Krings et al., 2014 (14)	Qazi et al., 2020 (15)	Koizumi et al., 2020 (16)
Clearly stated aim	2	2	2	2	2	2	2	2
Inclusion of consecutive patients	2	2	2	1	1	2	1	2
Prospective collection of data	0	0	2	1	0	0	1	0
Appropriate endpoints	2	2	2	2	2	2	2	2
Unbiased assessment of endpoints	1	0	1	1	1	1	0	1
Follow-up period appropriate	2	2	2	1	1	2	2	2
Loss to follow-up <5%	2	0	1	1	1	1	2	2
Prospective calculation of study size	0	0	0	0	0	0	0	0
Total	11/16	8/16	12/16	9/16	8/16	10/16	10/16	13/16

## Results

### Search results

After eliminating 1,196 duplicate entries, the systematic search yielded a total of 2,226 studies. The titles and abstracts of 1,030 studies were then carefully reviewed. Following this initial screening, 969 studies were excluded for not meeting the predefined criteria.

Of the 61 full reports that needed to be retrieved for further review, only four could not be located, leaving 57 papers to undergo a full-text assessment. Of these, 27 studies were excluded due to inaccurate or unreliable study results, 13 were removed because the population studied did not align with the focus of the review, 7 were categorized as editor's letters rather than original research, and 2 were rejected because they were abstracts lacking complete data.

**Figure 1:**
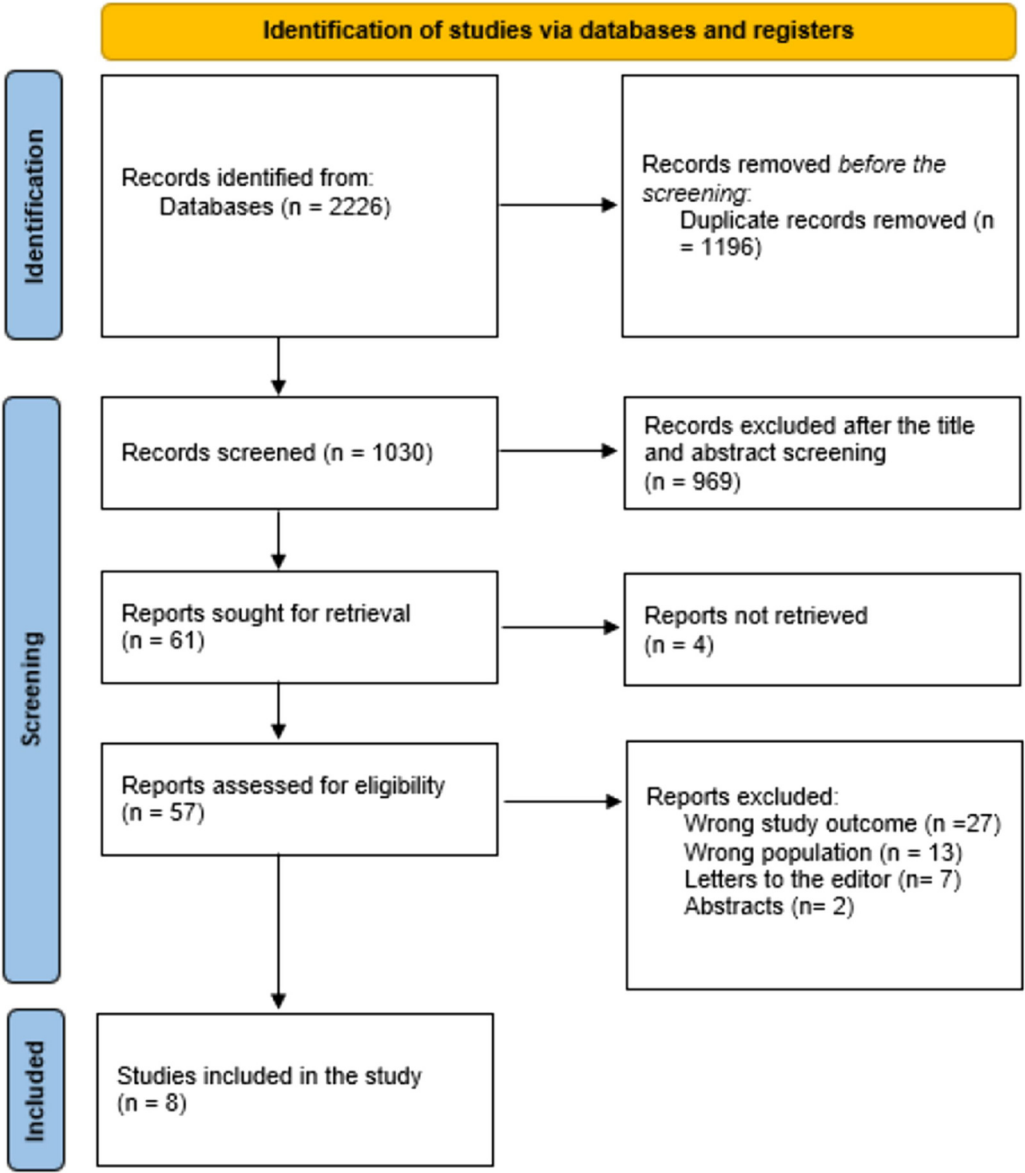
Study decision is summed up in a PRISMA diagram.

In the end, eight research studies successfully met all the eligibility criteria and were included in this systematic review. The entire process of study selection, from initial identification to final inclusion, is summarized in Figure 1.

### Sociodemographic features of the comprised studies

The sociodemographic characteristics of the included studies are summarized in Table 2. Our dataset included eight trials with a total of 204,286 patients, of which 118,567 (58%) were male. Six of the studies were retrospective cohort studies (1, 6, 11, 13, 14, 16), while two were prospective cohort studies (12, 15). Three studies took place in Japan (6, 12, 16), two in the USA (11, 14), and one each in India (16), Poland (1), and Saudi Arabia (13). The earliest study was from 2011 (11), and the most recent was from 2023 (13).

**Table 2:** Demographic characteristics of the included studies.

Study	Study design	Country	Participants	Mean age	Males (%)	Females (%)
Suzuki et al., 2015 (6)	Retrospective cohort	Japan	50,734	54 ± 15.4	33,191 (65.4%)	17,543 (34.6%)
Stankiewicz et al., 2011 (11)	Retrospective cohort	USA	105	49	56 (53.3%)	49 (46.7%)
Asaka et al., 2012 (12)	Prospective cohort	Japan	706	51.3 ± 15.8	483 (68.4%)	223 (31.6%)
Seredyka-Burduk et al., 2017 (1)	Retrospective cohort	Poland	1,658	45.6	987 (59.5%)	671 (40.5%)
Alharbi et al., 2023 (13)	Retrospective cohort	Saudi Arabia	1,831	36.12	871 (62.4%)	511 (36.60%)
Krings et al., 2014 (14)	Retrospective cohort	USA	78,944		39,302 (49.8%)	37,152 (50.2%)
Qazi et al., 2020 (15)	Prospective cohort	India	20	38.1	11 (55%)	9 (45%)
Koizumi et al., 2020 (16)	Retrospective cohort	Japan	70,288	55.6 ± 15.6	43,666 (62.1%)	26,622 (37.9%)

### Clinical outcomes

Clinical features are displayed in Table 3. Four studies performed FESS (6, 12, 14, 16), three performed ESS (11–13), and one performed image-guided ESS (15). All procedures were conducted for the management of CRS. The reported orbital complication rates varied substantially across the studies, ranging from 0% in the study utilizing image-guided ESS (15) to 27.6% in a study of conventional ESS (11). A total of 358 orbital complications were reported across all 204,286 patients. Due to the marked heterogeneity in study characteristics and complication definitions, a pooled incidence percentage is not reported; instead, the data are presented descriptively in Table 3. The most frequently reported orbital complications following sinus surgery were orbital injury, as identified by the ICD-10 codes for orbital hematoma (H052), disorder of binocular movement (H519), fracture of the orbital floor (S023), other orbital parts (S028) (1, 3, 6, 12, 16), orbital hematoma (11, 13), and orbital subcutaneous emphysema (11, 12).

**Table 3:** Clinical features of the included studies.

Study	Participants	Surgery	Condition	Orbital complications rate (%)	Orbital complications	Main outcomes
Suzuki et al., 2015 (6)	50,734	FESS	CRS	57 (0.09%)	Orbital injury (0.09%)	Compared to less comprehensive FESS, whole sinus surgery was not linked to increased incidence of ocular injury, CSF leakage necessitating surgery, or postoperative bleeding.
Stankiewicz et al., 2011 (11)	105	ESS	CRS	29 (27.6%)	Orbital hematoma (19%) Orbital subcutaneous emphysema (3.8%) Diplopia (0.9%)	Even after endoscopic sinus surgery was first introduced in 1985, complications might still arise from the procedure. Numerous issues are manageable with positive results. None of the patients experienced blindness directly related to the sinus surgery itself (e.g., from direct damage to the optic nerve or structures during surgery).
Asaka et al., 2012 (12)	706	ESS	CRS	38 (5.4%)	Minor orbital complication (2.0%) Subcutaneous periorbital emphysema (0.1%)	The polyp score and asthma were risk factors for postoperative complications.
Seredyka-Burduk et al., 2017 (1)	1,658	FESS	CRS	11 (0.7%)	Injury of lacrimal duct (0.06%) Injury of lacrimal duct (0.12%) Optic nerve injury (0.12%) Injury of orbital muscle (0.06%)	Rarely can endoscopic nasal surgeries result in orbital problems. Less than 0.3% of cases result in major problems that cause lifelong impairments.
Alharbi et al., 2023 (13)	1,831	ESS	CRS	2 (0.1%)	Orbital hematoma (0.1%)	ESS is a safe technique that frequently yields positive results with few complications this is due to advancements in technology, a deeper comprehension of anatomy and pathophysiology, and enhanced surgical training.
Krings et al., 2014 (14)	78,944	FESS	CRS	178 (0.23%)	Diplopia Paralytic stabismus Optic nerve injury Blindness Epiphora Orbital hemorrhage	The total significant complication rate following primary FESS is rather low, at 0.36%. Moreover, the total major complication rate following the FESS amendment is not statistically significant compared to that of the FESS originally.
Qazi et al., 2020 (15)	20	Image-guided ESS	CRS	0	No orbital complication was seen.	The ability to localize surgical tools in relation to pathology or important structures utilizing imaging in three dimensions is one advantage of image-guided surgery, which enhances surgeon confidence and safety for patients.
Koizumi et al., 2020 (16)	70,288	FESS	CRS	28 (0.04%)	Orbital injury 0.04% (orbital hematoma, a disorder of binocular movement, optic nerve injury, or orbital fracture)	The occurrence of general complications was not linked to the severity of FESS. The rate of total orbital injuries, both with and without the use of a microdebrider, was 0.04%. The use of a microdebrider did not show any significant connection to the occurrence of orbital injuries (*P* = 0.96).

The lowest rate of orbital complications (0%) was reported in the study utilizing image-guided ESS (15), whereas the highest rate (27.6%) occurred in a conventional ESS study (11). This variation highlights a correlation between surgical technique and reported outcomes, sample size, and study characteristics on the reported incidence of complications.

Data pertaining to risk factors and the management of orbital complications were extracted from the included studies (Table 3). The reported risk factors were primarily related to patient characteristics and surgical technique. Patient-specific risk factors included the presence of asthma and a high nasal polyp score (12). Regarding surgical factors, the use of image-guidance technology was correlated with lower reported complication rates, including a rate of 0% in one study (15). In contrast, conventional ESS techniques, particularly in smaller series, were associated with higher rates (11). The extent of surgery (complete sinus dissection) was not linked to an increased risk of orbital injury in a large database study (6). Furthermore, neither revision surgery (14) nor the use of a microdebrider (16) showed a statistically significant association with higher orbital complication rates in the studies that investigated them.

Management strategies were closely tied to the type and severity of the complication. For vision-threatening orbital hematoma, immediate intervention was emphasized, including surgical decompression (e.g., canthotomy/cantholysis, endoscopic drainage) and medical management to reduce intraorbital pressure (11, 13). In contrast, orbital emphysema was typically managed conservatively with observation, patient counseling to avoid nose blowing, and sometimes prophylactic antibiotics (11, 12).

## Discussion

Collaboration between ophthalmology and otolaryngology is essential for managing conditions such as orbital trauma, tumor surgery, drainage of subperiosteal abscesses, lacrimal duct disorders, optic nerve decompression, and orbital decompression (17). To the best of our knowledge, this is the first comprehensive review specifically focusing on orbital complications following sinus surgery.

Our synthesis suggests that risk factors are multifaceted. Patient-related factors such as asthma and severe nasal polyposis have been identified, likely due to their association with more extensive mucosal disease and intraoperative bleeding, which can obscure anatomical landmarks (12). However, the most significant and modifiable factors appear to be surgical. The absence of image-guidance emerged as a prominent technical risk factor, correlating with higher complication rates in our review and supporting its role in enhancing anatomical precision (15). Interestingly, other technical factors like the extent of surgery (full vs. limited dissection) and the use of powered instrumentation (microdebrider) were not independently linked to increased orbital risk in large-scale analyses (6, 16), suggesting that surgeon expertise and adherence to anatomical boundaries may outweigh these variables. Furthermore, revision surgery did not confer a statistically significant increase in major orbital complication risk compared to primary surgery in one large study (14), which may reflect improved techniques and heightened surgeon vigilance in complex cases.

We found that the reported orbital complication rates varied widely, from 0% in image-guided ESS (15) to 27.6% in conventional ESS (11), with 358 total events identified. This extreme variation underscores the necessity of a narrative, rather than quantitative, synthesis. The primary factor influencing this range appears to be surgical technique; image-guidance, which enhances anatomical precision, was associated with the lowest risk (15). Secondly, study design and sample size critically affect reported rates. The exceptionally high rate (27.6%) comes from a small, retrospective cohort (*n* = 105) where a few events heavily influence the percentage (11), a known phenomenon in surgical outcomes research (2). In contrast, large database studies (*n* > 50,000) reported rates between 0.04% and 0.23% (6, 14, 16). Finally, inconsistent definitions of what constitutes an “orbital complication” (e.g., including minor emphysema vs. only major hematoma) between studies further contribute to the heterogeneity.

Considering the methodological heterogeneity, we deliberately refrained from calculating a single pooled incidence rate. As noted in our synthesis, the reported rates are heavily influenced by study design. Large-scale administrative database studies, which provide robust data on rare, major events captured in billing codes (e.g., orbital injury), yield consistently low risk estimates (0.04%–0.23%) (6, 14, 16). These figures likely reflect the incidence of significant, coded complications in broad populations. Conversely, findings from smaller, dedicated clinical cohorts—where researchers can actively identify and report both major and minor complications (e.g., subcutaneous emphysema)—offer a different perspective, suggesting a higher overall incidence of orbital events when all severities are considered (11, 12). This distinction is crucial for interpretation: the former provides the best evidence for the risk of a significant orbital complication, while the latter offers a more complete picture of the total spectrum of orbital sequelae encountered in clinical practice, underscoring the importance of surgical vigilance regardless of the overall low statistical risk.

From a practical perspective, these findings carry important implications for surgical training and patient counseling. Structured training programs emphasizing detailed orbital anatomy, careful instrumentation near the lamina papyracea, and early recognition of orbital injury are essential to minimizing complications. Furthermore, the demonstrated protective role of image-guided surgery suggests that its use should be considered in complex cases, revision surgeries, or patients with distorted anatomy.

In terms of patient consent, although the absolute risk of orbital complications is low, the potential severity including vision-threatening outcomes necessitates transparent discussion during preoperative counseling. Informing patients about both the rarity and seriousness of these complications supports ethical shared decision-making and aligns with contemporary standards of patient-centered care. These findings have practical implications: the use of image-guided ESS should be considered in high-risk cases or revision surgeries to minimize complications, surgical trainees should receive targeted training on orbital anatomy and safety, and patients should be adequately informed about potential risks during the consent process.

Some key benefits of image-guided surgery (IGS) that have been highlighted include the ability to perform sagittal reconstructions using 3D imaging, which enhances patient safety, boosts surgeon confidence, and reduces the risk of serious complications in the brain or eye areas. This is especially useful in revision surgeries involving deformed nasal structures or missing critical landmarks. IGS also helps surgeons accurately locate their tools relative to important structures or problem areas. Additionally, it has been valuable as a teaching tool for residents and medical students. However, this study also found some downsides, including the high cost of the equipment, longer surgery times, the need for precise registration to ensure accurate navigation, and occasional errors in accuracy ranging from 1 to 1.5 mm (15).

Because of its close anatomical proximity to the paranasal sinuses and nose, the orbit is vulnerable to surgical injury; trauma to the lamina papyracea, orbital hemorrhage, diplopia, and blindness need to be considered. The surgeon should leave the eyes open during surgery and inspect them after it is completed to look for any possible serious orbital problems. The supporting nurse should be instructed to keep an eye on the bulb when the surgeon is working in close proximity to the orbit. The surgical assistant or nurse may notice early manipulations at the lamina papyracea or periorbita based on observations of eye movements (18).

In this study, the most frequently reported orbital complications following sinus surgery were orbital injury (1, 3, 12, 16), orbital hematoma (11, 13), orbital subcutaneous emphysema (11, 12), diplopia (11), and infection (16). Surgery ESS performed under local and topical anesthesia has raised some safety concerns because any damage to the orbital hematoma could cause the patient to lose their vision, and any damage to the periorbital area could cause them to react painfully. Therefore, while under general anesthesia, no such benefits are attainable (17).

The primary cause of orbital hemorrhage is penetration of the lamina papyracea, which can happen with or without damage to the periorbita. Lesions in the orbital veins that line the lamina papyracea are the most common cause of orbital hemorrhage; damage to the ethmoid arteries is less common (19).

An orbital hematoma may form rapidly if an injured anterior ethmoid artery withdraws into the orbit and presents as proptosis. Untreated orbital bleeding can cause blindness or at least momentary visual impairment due to an increase in orbital pressure (20). This is brought on by a decrease in the optic nerve's vascular supply, which is very susceptible to ischemia. Therefore, in order to avoid blindness or a permanent visual impairment, an orbital hematoma needs to be treated immediately.

A dangerous side effect of sinus surgery is diplopia, which can be caused by injury to the medial rectus and superior oblique muscles, two ocular muscles that are close to the sinuses. Most often injured is the medial rectus muscle, which is situated in the middle of the lamina papyracea and runs straight lateral to the periorbita. It is important to realize that the layer of intraorbital fat thins and offers less protection to the medial rectus muscle as one moves posteriorly. Damage to the medial rectus muscle can result in severe strabismus and unsettling diplopia, among other complications. The injury could have been caused by a direct cut of the muscle or by a neighboring nerve and vascular supply. Muscles harmed by machines seldom recover on their own (21, 22).

Fractures or perforations of the lamina papyracea resulting from sinus surgery can cause periorbital emphysema and ecchymosis. Periorbital emphysema can occur when a patient wakes up with a cough and blows their nose, allowing air to enter the soft tissues surrounding the orbit. The characteristic clinical indications of emphysema include a floppy eye bulb and rustling of the soft tissue in the infraorbital region, without a decrease in vision. Emphysema patients are treated with non-invasive breathing apparatuses and easy observation. The emphysema usually returns after seven to ten days (5).

Suzuki et al. reported that whole sinus surgery was not linked to increased incidence of ocular injury, CSF leakage necessitating surgery, or postoperative bleeding (6), while Asaka et al. mentioned that the polyp score and asthma were risk factors for postoperative complications (12).

Seredyka-Burduk et al. stated that endoscopic nasal surgeries can rarely result in orbital problems (1). Also, Alharbi et al. conclude that ESS is a safe technique that frequently yields positive results with few complications and that this is due to advancements in technology (13). Finally, Qazi et al. agreed with Alharbi et al. that the ability to localize surgical tools in relation to pathology or important structures utilizing imaging in three dimensions is one advantage of IGS, which enhances surgeon confidence and safety for patients (15).

Koizumi et al. concluded that the occurrence of general complications was not linked to the severity of FESS. The rate of total orbital injuries, both with and without the use of a microdebrider, was 0.04%. The use of a microdebrider did not show any significant connection to the occurrence of orbital injuries (*P* = 0.96) (16).

## Conclusion

ESS orbital complications are uncommon but have the potential to be dangerous. The reviewed evidence suggests an association between image-guided ESS and lower reported rates of orbital complications. Future prospective and randomized trials are required to determine the safest approach to ESS to avoid complications.

## Data Availability

The original contributions presented in the study are included in the article/Supplementary Material, further inquiries can be directed to the corresponding author.
